# Concordance of Text Message Ecological Momentary Assessment and Retrospective Survey Data Among Substance-Using Men Who Have Sex With Men: A Secondary Analysis of a Randomized Controlled Trial

**DOI:** 10.2196/mhealth.5368

**Published:** 2016-05-26

**Authors:** Christopher Rowe, Jaclyn Hern, Anna DeMartini, Danielle Jennings, Mathew Sommers, John Walker, Glenn-Milo Santos

**Affiliations:** ^1^ San Francisco Department of Public Health San Francisco, CA United States; ^2^ University of California, San Francisco San Francisco, CA United States

**Keywords:** data collection, cell phones, drug users, drinking behavior, homosexuality, male

## Abstract

**Background:**

Alcohol and illicit drug use is more prevalent among men who have sex with men (MSM) compared to the general population and has been linked to HIV transmission in this population. Research assessing individual patterns of substance use often utilizes questionnaires or interviews that rely on retrospective self-reported information, which can be subject to recall bias. Ecological momentary assessment (EMA) is a set of methods developed to mitigate recall bias by collecting data about subjects’ mental states and behaviors on a near real-time basis. EMA remains underutilized in substance use and HIV research.

**Objective:**

To assess the concordance between daily reports of substance use collected by EMA text messages (short message service, SMS) and retrospective questionnaires and identify predictors of daily concordance in a sample of MSM.

**Methods:**

We conducted a secondary analysis of EMA text responses (regarding behavior on the previous day) and audio computer-assisted self-interview (ACASI) survey data (14-day recall) from June 2013 to September 2014 as part of a randomized controlled trial assessing a pharmacologic intervention to reduce methamphetamine and alcohol use among nondependent MSM in San Francisco, California. Reports of daily methamphetamine use, alcohol use, and binge alcohol use (5 or more drinks on one occasion) were collected via EMA and ACASI and compared using McNemar’s tests. Demographic and behavioral correlates of daily concordance between EMA and ACASI were assessed for each substance, using separate multivariable logistic regression models, fit with generalized estimating equations.

**Results:**

Among 30 MSM, a total of 994 days were included in the analysis for methamphetamine use, 987 for alcohol use, and 981 for binge alcohol use. Methamphetamine (EMA 20%, ACASI 11%, *P*<.001) and alcohol use (EMA 40%, ACASI 35%, *P*=.001) were reported significantly more frequently via EMA versus ACASI. In multivariable analysis, text reporting of methamphetamine (adjusted odds ratio 0.06, 95% CI 0.04-0.10), alcohol (0.48, 0.33-0.69), and binge alcohol use (0.27, 0.17-0.42) was negatively associated with daily concordance in the reporting of each respective substance. Compared to white participants, African American participants were less likely to have daily concordance in methamphetamine (0.15, 0.05-0.43) and alcohol (0.2, 0.05-0.54) reporting, and other participants of color (ie, Asian, Hispanic, multi-racial) were less likely to have daily concordance in methamphetamine reporting (0.34, 0.12-1.00). College graduates were more likely to have daily concordance in methamphetamine reporting (6.79, 1.84-25.04) compared to those with no college experience.

**Conclusions:**

We found that methamphetamine and alcohol use were reported more frequently with daily EMA texts compared to retrospective ACASI, concordance varied among different racial/ethnic subgroups and education levels, and reported substance use by EMA text was associated with lower daily concordance with retrospective ACASI. These findings suggest that EMA methods may provide more complete reporting of frequent, discrete behaviors such as substance use.

## Introduction

Use of alcohol and illicit drugs (ie, substance use) is a major contributor to the global burden of disease [1,2] and is more prevalent among men who have sex with men (MSM) than among the general population [3,4]. In a 2011 national sample of MSM across 20 US cities, 85% reported drinking alcohol and 50% reported binge drinking (having 5 or more alcoholic beverages on a single occasion) in the past 30 days and 49% reported using noninjection drugs in the past 12 months [3]. Heavy alcohol use and the use of noninjection drugs, particularly methamphetamine, have been linked to the transmission and acquisition of HIV among MSM, who continue to be more affected by HIV than any other demographic group in the United States [5]. Understanding alcohol and substance use behaviors among MSM at high risk for HIV is essential for effectively informing efforts to reduce HIV transmission and improve overall health in this population.

Research assessing individual patterns and the frequency of alcohol and substance use often utilizes questionnaires or interviews that rely on retrospective self-reported information. A major limitation of these methods is that information recalled from memory is prone to distortion, which can affect the reliability of collected data [6,7]. Ecological momentary assessment (EMA) is a set of methods developed to mitigate recall bias by collecting data about subjects’ mental states and behaviors on a near real-time basis [7,8]. EMA methods involve repeated assessments within subjects’ natural environments and routines and vary widely in design [9]. Studies using EMA methods, for example, may combine participant-initiated data reporting with randomly administered assessments throughout the day, or administer assessments at regular intervals to achieve abbreviated recall windows ranging from hours to an entire day [9-13]. Moreover, these methods can employ written or electronic diaries, physiological sensors, or mobile devices, such as mobile phones [7]. The popularity and near-universal prevalence of mobile phones makes them a powerful conduit for data collection, especially with the simplicity of text messaging (short message service, SMS) [14]. Furthermore, EMA may be particularly effective for substance use research due to the episodic and discrete nature of substance use behavior, which makes it better suited for real-time data collection than behaviors or states that are more ongoing [9]. Despite concerns regarding the ability and willingness of substance-using populations to adhere to the high-engagement requirements of EMA methods, a growing body of literature has demonstrated the feasibility, acceptability, and reliability of EMA among those who engage in substance use [15-21].

Although it has shown to be successful, EMA remains underutilized in substance use and HIV research. To date, relatively few studies have employed EMA methods to assess substance use patterns among MSM, and none have assessed the concordance of information reported using EMA with retrospective questionnaires among this population. To better understand the utility of different data collection methods among substance-using MSM, we aimed to assess the concordance between daily reports of substance use collected by EMA text messages and retrospective questionnaires, and to identify predictors of daily concordance.

## Methods

### Study Sample

We conducted a secondary analysis of EMA text responses and audio computer-assisted self-interview (ACASI) survey data from June 2013 to September 2014 as part of a randomized controlled trial assessing the feasibility, acceptability, and tolerability of a pharmacologic intervention to reduce methamphetamine and alcohol use among nondependent MSM in San Francisco, California [22]. Active recruitment efforts included outreach at municipal sexually transmitted infection (STI) and HIV clinics, MSM community-based organizations, gay bars and events, and syringe access programs. Passive recruitment efforts included flyers at active recruitment sites and advertisements in local newspapers, gay print media, and on social media. Eligibility criteria included: self-reported methamphetamine use at least 2 times per month, binge alcohol use at least once per week, methamphetamine or alcohol use concurrent with anal intercourse in the past 3 months, and a desire to reduce or discontinue methamphetamine and alcohol use. Additionally, participants were required to have a mobile phone that could send and receive text messages. All study participants provided informed consent and study procedures were approved by the Committee on Human Research, University of California, San Francisco.

### Data Collection

EMA data were collected via daily text messages. Beginning the day after a baseline visit, participants were sent daily text messages asking about their behaviors on the previous day. They were asked whether they had taken the study drug, used methamphetamine, or used alcohol (and if so, the number of alcoholic drinks consumed). Participants received this text message series every day until the conclusion of the follow-up period (approximately 2 months). As reimbursement for their time, participants received a $1 stipend for each day that they completed the text message series.

ACASI surveys were administered at baseline and approximately every 2 weeks for a total of 5 visits. Each ACASI survey asked participants whether or not they had engaged in the following behaviors on each of the preceding 14 days: methamphetamine use, alcohol use, and binge alcohol use. To enhance recall, participants were provided with the exact calendar dates of the preceding 14 days as part of the assessments for these 3 behaviors, using a modified timeline follow-back approach.

Additional data collected at baseline included: demographic characteristics, including race, age, and highest level of education achieved; number of days in the past 4 weeks during which the participant engaged in binge alcohol use; and the frequency of methamphetamine use in the past 4 weeks as either less than one day per month or a selection of 1 through 4 days per month and 2 through 7 days per week. The frequency of methamphetamine use was then converted into a continuous variable to correspond to the number of days in the past 4 weeks during which methamphetamine was used. Due to our small sample size and the limited number of participants who identified as a race other than white or African American (Latino/Hispanic n=5, Asian American or Pacific Islander n=2, mixed or multi-racial n=2), participants who were neither white nor African American were combined into an “other” race category.

### Reporting and Concordance Measures

In the analysis for each substance, days were only included for which both an ACASI recall response and an EMA text response were present. Such that, if a participant did not complete the text series on a given day, did not complete an ACASI, or if study visits were scheduled more than 14 days apart (resulting in gaps in ACASI recall), concordance could not be assessed due to missing data. These days were therefore excluded in the concordance analysis.

Pooled for the entire sample, the total number and proportion of days on which methamphetamine use, alcohol use, and binge alcohol use were reported were calculated for both ACASI and EMA. McNemar’s tests were used to assess differences in reported frequencies of methamphetamine, alcohol, and binge alcohol use between EMA text and ACASI modified timeline follow-back data.

The total number and proportion of days for which ACASI and EMA responses matched for each substance (ie, concordant responses) were calculated. This included both concordant positive responses (ie, days that methamphetamine use, alcohol use, or binge alcohol use were reported through both ACASI and EMA) and concordant negative responses (ie, days that no methamphetamine use, no alcohol use, or no binge alcohol use was reported through both ACASI and EMA). We also calculated the phi coefficient, which is equivalent to Pearson’s correlation coefficient when applied to binary variables, between ACASI and EMA responses.

### Multivariable Analysis

For our primary multivariable analysis, we used multivariable logistic regression models, fit with generalized estimating equations that accounted for clustering within-subject, to assess the likelihood of having concordant responses on a given day for methamphetamine use, alcohol use, and binge alcohol use. All models included demographic and behavioral characteristics as time-invariant covariates and the number of days elapsed since the baseline visit as a time-varying covariate. Overall, 3 models (one for each substance-specific concordance outcome) included a time-varying binary covariate indicating whether or not the relevant substance was reported by EMA text on the day being assessed. Each substance-specific model included only the time-varying EMA text response covariate for the substance being assessed in that model (ie, methamphetamine, alcohol, or binge alcohol).

In sensitivity analyses, 3 alternative models (one for each substance-specific concordance outcome) included a covariate indicating the number of alcohol beverages reported by EMA text on the day being assessed. In the model assessing concordance of methamphetamine use reporting, both the binary covariate indicating any methamphetamine use and the continuous covariate indicating the number of drinks consumed were included. In the models assessing concordance of alcohol and binge alcohol use reporting, only the covariate indicating the number of drinks consumed was included. All other covariates in these 3 models were the same as in the primary models.

## Results

### Study Sample Characteristics

As presented in Table 1, our study sample of 30 participants was racially/ethnically diverse: 12 participants (40%) were white, 9 (30%) were African American, and 9 (30%) identified as any other race/ethnicity. The mean age was 43 (SD 9.3) and 26 (87%) had completed at least some college education, with 9 (30%) having a Bachelor’s degree or higher. The average number of days of substance use in the 4 weeks prior to baseline was 6 (SD 4.6) for methamphetamine, 13 (SD 7.6) for alcohol, and 7 (SD 7.2) for binge alcohol.

**Table 1:** Demographic and substance use characteristics of study participants (n=30).

		n (%) or mean (SD)
Age, mean (SD)		43 (9.3)
Race	White	12 (40.0)
	African American	9 (30.0)
	Other	9 (30.0)
Education	High school diploma or less	4 (13.3)
	Some college	17 (56.7)
	Bachelor's degree or higher	9 (30.0)
Days of Substance Use in Last 4 Weeks at Baseline	Methamphetamine, mean (SD)	6 (4.6)
	Alcohol, mean (SD)	13 (7.6)
	Binge alcohol, mean (SD)	7 (7.2)

### ACASI and EMA Text Compliance

All compliance measures are reported in Table 2. In total, 30 participants completed 143 (95%) biweekly ACASI surveys out of the intended 150. Twenty-four participants (80%) completed all 5 intended surveys, 5 participants (17%) completed 4 surveys, and 1 participant (3%) completed 3 surveys. Because initiation of daily text messages did not begin until the day after the participants’ first ACASI (with the exception of 1 participant who initiated daily text messages early and provided 4 days of EMA recall prior to their first ACASI), all but these 4 recall responses from participants’ first ACASI surveys were not relevant to this analysis. Across the 30 participants and including the aforementioned 4 additional days, a total of 1469 (mean per participant 49, SD 7.7) days of methamphetamine use reporting and 1462 (mean per participant 49, SD 7.9) days of alcohol and binge alcohol use reporting were collected via participants’ noninitial ACASI surveys.

Of the 1469 and 1462 days of ACASI reporting for methamphetamine and alcohol/binge alcohol use, respectively, EMA text responses were collected on 994 days (68%) for methamphetamine use, 987 (68%) for alcohol use, and 981 (67%) for binge alcohol use. For each substance, EMA text reporting was not available for 156 (11% for each substance) days due to delayed initiation or technical issues and 319 (22% for each substance) days due to participant nonresponse. Of days for which ACASI reporting was available, the average number of days of EMA text data available was 33.1 for methamphetamine use (range 10-55, SD 13.8), 32.9 for alcohol use (range 10-55, SD 13.8), and 32.7 for binge alcohol use (range 10-55, SD 13.9). As a percentage of the days of ACASI data per participant, these numbers correspond to 67.2% for methamphetamine use (range 19-98, SD 24.2), 67.1% for alcohol use (range 19-98, SD 24.1), and 66.6% for binge alcohol use (range 19-98, SD 24.2).

### Reporting and Concordance Measures

**Figure 1:**
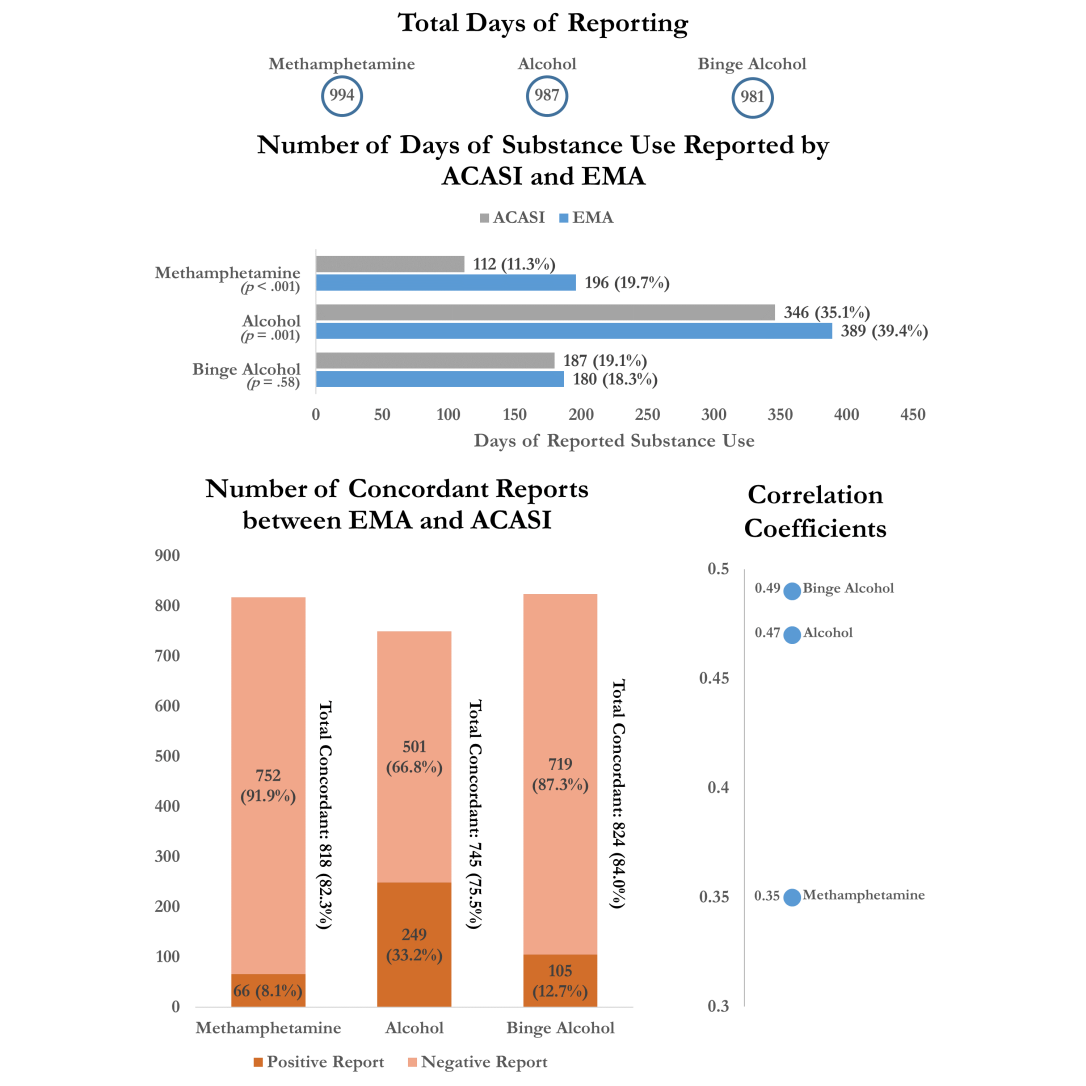
Reporting and concordance measures for entire pooled sample (n = 30).

A total of 994 days were included in the analysis for methamphetamine use, 987 for alcohol use, and 981 for binge alcohol use (see Figure 1). There were significant differences in the proportion of days on which methamphetamine and alcohol use were reported by text versus ACASI (*P*<.05). The frequencies reported by EMA text and ACASI, respectively, were 20% and 11% for methamphetamine use (*P*<.001) and 39% and 35% for alcohol use (*P*=.005). There was no significant difference in the proportion of days on which binge alcohol use was reported by text versus ACASI (*P*=.58). Of the concordant responses for each substance, the majority were reports of no substance use (92% for methamphetamine use, 67% for alcohol use, and 87% for binge alcohol use). The phi coefficients were 0.35 for methamphetamine use, 0.49 for alcohol use, and 0.47 for binge alcohol use.

**Table 2:** ACASI and EMA text message reporting compliance.

			n (%) or mean (range, SD)
ACASI Compliance	Overall Compliance of Biweekly ACASI Surveys	Total number intended	150
		Total number completed	143 (95.3)
	Number of ACASI Surveys Completed by Study Participants (n=30)	5 (100% compliance)	24 (80.0)
		4 (80% compliance)	5 (16.7)
		3 (60% compliance)	1 (3.3)
	Total Number of Days of ACASI Data^a^	Methamphetamine use	1469
		Alcohol use	1462
		Binge alcohol use	1462
	Number of Days of ACASI Data Per Participant^a^	Methamphetamine use mean (range, SD)	49 (28-56, 7.7)
		Alcohol use mean (range, SD)	49 (28-56, 7.9)
		Binge alcohol use mean (range, SD)	49 (28-56, 7.9)
EMA Text Message Compliance^b^	Methamphetamine Use Reporting (Pooled for Entire Sample)	Complete text response	994 (67.7)
		Missing text response	319 (21.7)
		Delayed initiation or system issue	156 (10.6)
	Alcohol Use Reporting (Pooled for Entire Sample)	Complete text response	987 (67.5)
		Missing text response	319 (21.8)
		Delayed initiation or system issue	156 (10.7)
	Binge Alcohol Use Reporting (Pooled for Entire Sample)	Complete text response	987 (67.5)
		Missing text response	319 (21.8)
		Delayed initiation or system issue	156 (10.7)
	Number of Days of EMA Text Data Per Participant	Methamphetamine use mean (range, SD)	33.1 (10-55, 13.8)
		Alcohol use mean (range, SD)	32.9 (10-55, 13.8)
		Binge alcohol use mean (range, SD)	32.7 (10-55, 13.9)
	Days of EMA Text Data as Percentage of Days of ACASI Data Per Participant	Methamphetamine use mean (range, SD)	67.2 (19-98, 24.2)
		Alcohol use mean (range, SD)	67.1 (19-98, 24.1)
		Binge alcohol use mean (range, SD)	66.6 (19-98, 24.2)

^a^With the exception of 4 days for a single participant, recall responses from participants' first ACASI survey were not relevant to this analysis because EMA text messaging did not begin until the day after the first survey was completed. These are excluded from the total number of days of ACASI data available.

^b^EMA text message compliance was assessed as the proportion of the number of days of ACASI data available.

### Multivariable Analysis

The results of the generalized estimating equation fitted multivariable logistic regression models are presented in Tables 3-6. In the primary model assessing methamphetamine reporting (Table 3), text reporting of any methamphetamine use was negatively associated with daily concordance in methamphetamine reporting (adjusted odds ratio 0.06, 95% CI 0.04-0.10). African American (0.15, 0.05-0.43) and other participants of color (ie, Hispanic/Latino, Asian American and Pacific Islander, mixed or multi-racial individuals) (0.34, 0.12-1.00) were also less likely to have daily concordance in methamphetamine reporting compared to white participants. Participants who had graduated from college had a greater odds of methamphetamine reporting concordance (6.79, 1.84-25.04) compared to those with no college experience. In the sensitivity analysis that included both the text reports of any methamphetamine use and number of alcoholic drinks, number of drinks was not independently associated with methamphetamine reporting concordance and the coefficient estimates of all other covariates were not significantly different than those in the primary model (*P*=0.69, data not shown).

In the primary model assessing alcohol reporting (Table 4), text reporting of any alcohol use was negatively associated with daily concordance in reporting of alcohol use (0.50, 0.34-0.73). African American participants were less likely to have daily concordance in alcohol use reporting compared to white participants (0.18, 0.06-0.57). In the sensitivity analysis that included the number of alcoholic drinks reported by text, number of drinks was not independently associated with alcohol reporting concordance and coefficient estimates of the other covariates were not significantly different than those in the primary model (*P*=0.11, data not shown).

In the primary model assessing binge alcohol reporting (Table 5), text reporting of any binge alcohol use was negatively associated with daily concordance in reporting of binge alcohol use (0.27, 0.17-0.42). In the sensitivity analysis that included the number of alcoholic drinks reported by text (Table 6), number of drinks was negatively associated with concordance in binge alcohol use reporting (0.89, 0.84-0.94). Also, in this model, African American participants were less likely to have daily concordance in binge alcohol use reporting compared to white participants (0.25, 0.07-0.87).

**Table 3:** Multivariable logistic regression model, fit with generalized estimating equations, assessing daily EMA-ACASI concordance in methamphetamine reporting (n=994).

Variable		Daily EMA-ACASI Concordance in Methamphetamine Reporting		
		OR	(95% CI)	*P*-value
Text Report of Any Methamphetamine Use		0.06	(0.04-0.10)	<.001
Race	White	Reference		
	African American	0.15	(0.05-0.43)	<.001
	Other^a^	0.34	(0.12-1.00	.049
Age		1.02	(0.97-1.08)	.46
Education	High school graduate or less	Reference		
	Some college	2.17	(0.81-5.80)	.12
	College graduate or more	6.79	(1.84-25.04)	.004
Baseline Days of Meth Use in Past 4 Weeks		1.01	(0.92-1.10)	.88
Baseline Days of Binge Drinking in Past 4 Weeks		0.98	(0.93-1.04)	.52
Day of Follow-Up		1.00	(0.99-1.02)	.55

^a^Includes Hispanic/Latino, Asian American or Pacific Islander, and mixed or multi-racial individuals.

**Table 4:** Multivariable logistic regression model, fit with generalized estimating equations, assessing daily EMA-ACASI concordance in alcohol reporting (n=987).

Variable		Daily EMA-ACASI Concordance in Alcohol Reporting		
		OR	(95% CI)	*P*-value
Text Report of Any Alcohol Use		0.50	(0.34-0.73)	<.001
Race	White	Reference		
	African American	0.18	(0.06-0.57)	.004
	Other^a^	0.43	(0.17-1.08)	.07
Age		1.02	(0.97-1.07)	.40
Education	High school graduate or less	Reference		
	Some college	0.79	(0.27-2.32)	.67
	College graduate or more	1.17	(0.31-4.36)	.81
Baseline Days of Meth Use in Past 4 Weeks		1.08	(0.99-1.18)	.07
Baseline Days of Binge Drinking in Past 4 Weeks		1.03	(0.96-1.10)	.38
Day of Follow-Up		1.02	(1.01-1.03)	<.001

^a^Includes Hispanic/Latino, Asian American or Pacific Islander, and mixed or multi-racial individuals.

**Table 5:** Multivariable logistic regression model, fit with generalized estimating equations, assessing daily EMA-ACASI concordance in binge alcohol reporting (with text report of any binge alcohol use predictor) (n=981).

Variable		Daily EMA-ACASI Concordance in Binge Alcohol Reporting		
		OR	(95% CI)	*P*-value
Text Report of Any Binge Alcohol Use		0.27	(0.17-0.42)	<.001
Race	White	Reference		
	African American	0.35	(0.11-1.31)	.08
	Other^a^	0.69	(0.22-2.18)	.53
Age		1.02	(0.97-1.09)	.41
Education	High school graduate or less	Reference		
	Some college	1.68	(0.52-5.38)	.39
	College graduate or more	2.67	(0.64-11.1)	.18
Baseline Days of Meth Use in Past 4 Weeks		1.05	(0.95-1.17)	.30
Baseline Days of Binge Drinking in Past 4 Weeks		0.97	(0.91-1.03)	.33
Day of Follow-Up		1.01	(1.00-1.02)	.26

^a^Includes Hispanic/Latino, Asian American or Pacific Islander, and mixed or multi-racial individuals.

**Table 6:** Multivariable logistic regression model, fit with generalized estimating equations, assessing daily EMA-ACASI concordance in binge alcohol reporting (with text report of number of drinks predictor) (n=981).

Variable		Daily EMA-ACASI Concordance in Binge Alcohol Reporting		
		OR	(95% CI)	*P*-value
Text Report of Number of Drinks		0.89	(0.84-0.94)	<.001
Race	White	Reference		
	African American	0.25	(0.07-0.87)	.03
	Other^a^	0.68	(0.22-2.11)	.50
Age		1.05	(0.99-1.11)	.14
Education	High school graduate or less	Reference		
	Some college	1.40	(0.45-4.34)	.56
	College graduate or more	2.29	(0.55-9.54)	.26
Baseline Days of Meth Use in Past 4 Weeks		1.07	(0.97-1.19)	.16
Baseline Days of Binge Drinking in Past 4 Weeks		0.98	(0.91-1.04)	.46
Day of Follow-Up		1.01	(0.99-1.02)	.35

^a^Includes Hispanic/Latino, Asian American or Pacific Islander, and mixed or multi-racial individuals.

## Discussion

### Principal Findings

We found that substance use was reported more frequently using daily text messaging compared to retrospective questionnaires, with the greatest differential in the reporting of methamphetamine use. Daily concordance between EMA and questionnaire self-reports was not homogenous across participants; specifically, congruent reports were less likely among non-white participants and more likely among those who had graduated from college. Lastly, on days in which each substance was reported to have been used via EMA text response, use was significantly less likely to be reported in the ACASI questionnaire. These findings have implications for both the design and interpretation of substance use research among MSM.

In general, EMA captured more frequent substance use than ACASI. For example, methamphetamine use was reported nearly twice as frequently and alcohol use roughly 14% more frequently via EMA as compared to ACASI. Participants in our study were not compensated differentially based on their EMA responses; in addition, EMA text exchanges could be expedited by reporting no substance use. Therefore, participants had no material or time-related incentive to over-report their substance use by EMA text. Both ACASI and EMA may minimize concerns related to social desirability bias through the use of electronic, self-administered questionnaires as opposed to in-person interviews [23,24]. However, the frequent, repeated assessments of EMA methods are designed to minimize the duration of participant recall and reliance on autobiographical memory, which can be distorted even after relatively short intervals [6,7]. For these reasons, we hypothesize that daily EMA texting may provide a more accurate and reliable assessment of substance use behaviors compared to questionnaires with longer recall periods, such as the ACASI, or traditional in-person interviews. In addition to potential advantages related to data quality, EMA methods can ease the burden of data collection on study participants by allowing them to report information conveniently and privately during their daily routines and using their own personal devices.

In our multivariable analysis, participants of color were less likely to report concordantly between EMA and ACASI, for both methamphetamine and alcohol use. In prior studies comparing self-reported data to biological tests of substance use, African Americans were more likely to underreport substance use [25-29]. This may be the result of either a lower level of trust and greater reluctance among African Americans to disclose sensitive information to someone of different race, or cultural variations in the interpretation of survey questions [30-32]. African American participants also reported a higher frequency of substance use by EMA text compared to ACASI, suggesting that text-based EMA methods may be more acceptable to this population and may help to mitigate any systematic underreporting among this racial/ethnic subgroup.

We also found that the likelihood of daily concordance was significantly less for days during which participants reported substance use by text. In the case of binge alcohol use, likelihood of daily concordance also decreased as the number of drinks reported by text increased. These findings could be a function of either the acute and residual effects of each substance (as suggested by the association between number of drinks and binge alcohol use reporting concordance) or a greater difficulty in recalling exact temporal details of behaviors that occur more frequently. Although we were not able to assess the validity of our self-reported measures, several studies have demonstrated the validity of EMA reporting in relation to objective biochemical markers of cocaine, tobacco, and alcohol use [17,33,34]. If the EMA-collected data were an accurate representation of substance use behaviors, this finding suggests vulnerabilities with ACASI-collected substance use data in analyses where substance use is the exposure or outcome of interest. In this case, accurate recall and reporting of substance use behaviors would differentially depend on whether or not the participant actually used the substance, which would systematically bias any analytic findings (ie, recall bias) [35].

### Limitations

This study has several limitations. First, our small sample size limits both our statistical power to identify significant correlates of daily reporting concordance and our ability to explore differences across a wider array of racial/ethnic groups. Secondly, our convenience sample may not be generalizable to all substance-using MSM. We also had no day-to-day objective measures against which to validate either the EMA or ACASI self-reported measures of substance use; therefore, we cannot make conclusions as to the validity of either method. In addition, daily assessments of the number of drinks consumed in the previous day have been shown to elicit lower reported quantities compared to real-time hourly reporting [36]. Thus, although our assessments are mainly concerned with any substance use on a given day as opposed to the specific quantity consumed, our daily text message measure may be affected by recall bias. Finally, our analyses were restricted to days for which both EMA and ACASI data were available, which may have biased our results if EMA nonresponses or missed ACASI interviews were associated with substance use and likelihood of concordance. Although there were high rates of compliance for ACASI Surveys (95% overall), overall text compliance was modest (68% overall for each substance). An assessment of predictors of EMA text message engagement among the current study sample found that white participants had higher response rates than participants of color, and those who had at least some college education were less likely to stop responding to texts for a prolonged period [37]. This variability in compliance among different demographic groups may further affect the generalizability of the data assessed in this analysis. It is important for future studies to assess the characteristics, concordance, and validity of these data collection methods among samples with elevated reporting compliance.

### Conclusions

Our findings have important implications for the collection of self-reported data in substance use research, particularly among MSM. We found that methamphetamine and alcohol use were reported more frequently with daily EMA text compared to retrospective ACASI, concordance varied among different racial/ethnic subgroups and level of education, and reported substance use in EMA text was associated with lower daily concordance with retrospective ACASI. These findings suggest that EMA methods may provide more complete reporting about frequent, discrete behaviors such as substance use, which may be particularly valuable among subgroups more likely to underreport such behaviors.

## Abbreviations

MSM: men who have sex with men
EMA: ecological momentary assessment
ACASI: audio computer-assisted self-interview
STI: sexually transmitted infection
